# HOME HEALTH CARE ACCESS AND OUTCOMES AMONG VULNERABLE OLDER POPULATIONS: CHALLENGES AND OPPORTUNITIES

**DOI:** 10.1093/geroni/igae098.2153

**Published:** 2024-12-31

**Authors:** Emily Gadbois, Michael Johnson

**Affiliations:** Chair; Discussant

## Abstract

Home health care (HHC) provides skilled nursing (e.g., wound care, medication management), therapies (e.g., physical, occupational, speech), and aide services (e.g. bathing, grooming, dressing), which are key supports necessary to regain function, restore health, and maintain independence in the community. This panel presents five studies examining factors that challenge equitable access and high quality outcomes associated with skilled HHC among vulnerable older populations (i.e., Veterans, people living with dementia, patients needing post-acute care). The first panelist will discuss findings using national-level HHC quality data demonstrating that access to high-quality HHC varies widely across the country for people with dementia. The second panelist will share findings from a novel data linkage using Medicare Advantage (MA) and traditional Medicare claims to understand variation in use of HHC among people living with dementia. The third panelist’s presentation will focus on a particularly vulnerable population - hospitalized older Veterans - and how payer source shapes the use of post-acute HHC. The fourth panelist will discuss results from a qualitative study with MA plans about how they design and manage post-acute HHC benefits. The final presenter will share results from an analysis of national standardized HHC assessment data to identify failures in information transfer that impact care delivery and quality for HHC patients living with dementia. The discussant, a national industry leader with over two decades of experience working in the delivery of HHC, will contextualize findings and discuss how they can inform national policy discussions around improving access and quality of HHC.

